# The ASCO University Website: Online Education for the Oncology Advanced Practitioner

**DOI:** 10.6004/jadpro.2013.4.3.8

**Published:** 2013-05-01

**Authors:** Heather M. Hylton

**Affiliations:** Heather M. Hylton is a physician assistant at Memorial Sloan-Kettering Cancer Center in New York City.

Many clinicians rely upon electronic resources for information pertinent to the care they provide for their patients. While there are numerous resources to choose from, the selection process can be daunting because of the sheer volume of websites in existence and questions about the accuracy of the information presented on these websites, to name just a few variables.

The American Society of Clinical Oncology (ASCO) family of websites can equip the clinician with many valuable tools and references. The ASCO University, ASCOConnection, Cancer.Net, ASCO in Action, and main ASCO websites will be reviewed in a multipart series; the ASCO University website will be reviewed here.

## Overview of the ASCO University Website

Recently revamped, the ASCO University website (http://university.asco.org/) is a distinguished online education resource for professionals working in oncology. The home page features a menu bar from which one can easily access the principal areas of the site, including the course catalog and the bookstore, with a single click of the mouse. Also included on the home page are icons representing the latest additions to the ASCO University website along with featured courses.

When one clicks on "Course Catalog" from the home page, the user is directed to a page on the website titled "Cancer Topics" (http://university.asco.org/cancer-topics). Located just under the main menu bar on this page is another menu bar that highlights and contains links to different ASCO products and product lines, including the e-Seminars and Tumor Boards.

Under the "Cancer Topics" heading, the user will find multiple electronic tracks organized by tumor type or special topics. Each track is represented by a colorful icon and a track title such as "Breast Cancer" or "Clinical Trials." When one clicks on a track icon such as "Breast Cancer," ASCO University content that is related to breast cancer is displayed, with each content item indicated by its own icon. Overlap of content may be seen across the electronic tracks based upon the subject matter.

## Identifying Content of Interest

Numerous topic areas and product lines that may be of interest to the advanced practitioner in oncology can be found on the ASCO University website. While by no means a comprehensive listing of what is available on the site, the following may serve as a helpful guide to navigating site content. A number of the courses that follow also feature advanced practice provider faculty.

**Focus Under Forty** (http://university.asco.org/focus-under-forty) is a series of online educational courses, jointly developed with multiple education partners including ASCO and LIVE**STRONG**, geared toward recognizing and addressing the needs of the adolescent and young adult patient population with cancer. Courses in this educational program include Breast Cancer as a Second Malignancy, Cancer Risk Reduction Review, and Survivorship, to name just a few. Included in the course is a case presentation with audio commentary, a full bibliography, select ASCO Virtual Meeting presentations, and a listing of resources for patients. The Focus Under Forty courses are available at no cost, and Category 1 CME credit is available.

**ASCO Tumor Boards** (http://university.asco.org/e-learning/tumor-boards) are a series of online case-based multidisciplinary courses spanning a broad spectrum of topics including Thyroid Cancer, Glioblastoma, and Carcinoma of Unknown Primary. Included in the course is a case presentation with audio commentary, a full bibliography, select ASCO Virtual Meeting presentations, and a listing of resources for patients. These evidence-based presentations can be accessed on the ASCO University website at no cost, and a certificate is provided upon successful completion of the educational program.

**e-Seminars** (http://university.asco.org/e-seminars-0) are captures of previously held live web-based seminar events. A broad scope of topics is addressed through the e-Seminars including Team-Based Care in Oncology, Annual Meeting Highlights (available from multiple years), and Quality Improvement: Getting Started. Included in each course is a PDF version of the slides presented in the live event, a full bibliography, select ASCO Virtual Meeting presentations, and a listing of resources for patients. These courses are available for a small fee, and all of the e-Seminar capture courses provide a certificate upon successful completion of the educational program; Category 1 CME credit is available for a limited number of courses.

**Symptom Management topics** can be found by clicking on the "Patient & Survivor Care" icon under the Course Catalog tab (http://university.asco.org/e-learning/cancer-topics/34). The courses featured in this series of topics include Chemotherapy Related Nausea and Vomiting, Cancer Related Fatigue, and Cancer Pain. Included in each course is an online presentation with audio commentary, a listing of course references, and a listing of resources geared toward patients. These courses are available for a small fee, and Category 1 CME credit is available.

**Topics on Quality** can also be found when clicking on the "Patient and Survivor Care" icon under the Course Catalog tab (http://university.asco.org/e-learning/cancer-topics/34). The courses featured in this series of topics include Chemotherapy Safety Standards and Engaging in Quality Improvement. Each course includes a case presentation with audio commentary, course references, select ASCO Virtual Meeting presentations, and a listing of resources geared toward patients. These courses are available for a small fee, and Category 1 CME credit is available.

**Oncology Literature Reviews** (http://university.asco.org/oncology-literature-reviews-past-issues) are online courses that highlight and summarize papers recently published in the Journal of Clinical Oncology and other major journals on the topic areas of lymphoma, breast, gastrointestinal, genitourinary, and lung cancers. These literature reviews are released on a quarterly basis, and included in each course is a case presentation with audio commentary and course references. These courses are available for a small fee, and all of the courses provide a certificate upon successful completion of the educational program.

**ASCO-SEP or Self-Evaluation Program** (http://university.asco.org/asco-sep-third-edition), now in its third edition, is a comprehensive self-study program available in print and electronic formats (see Figure). Products that complement the ASCO-SEP are also available and include an online question bank and a flashcard application. Additionally, the ASCO-SEP Companion Series (http://university.asco.org/exam-preparation) is available and features special topics for self-evaluation including reviews in palliative care, biostatistics, and disease-focused reviews, to name a few.

**Figure 1:**
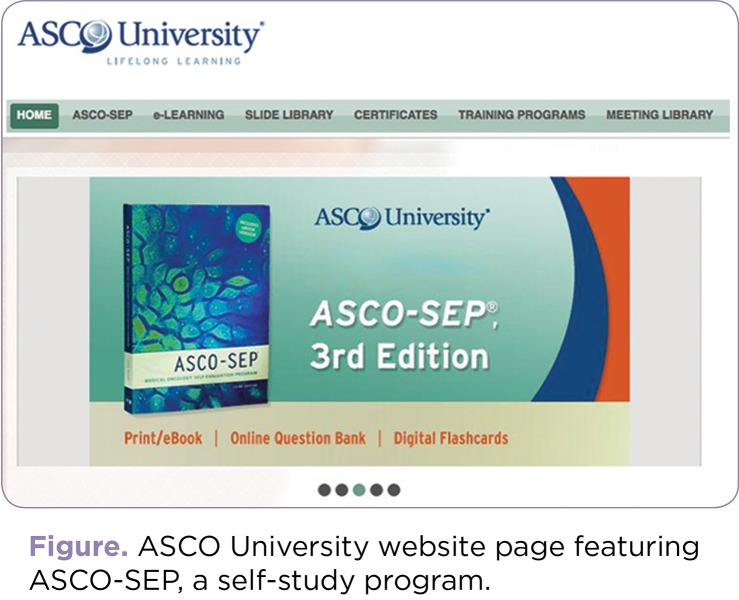
Figure. ASCO University website page featuring ASCO-SEP, a self-study program.

There are many additional offerings on the ASCO University website, including population-specific courses, the Oncology Slide Library (http://university.asco.org/slide-library), the Meeting Library (http://meetinglibrary.asco.org/), and the ASCO University Bookstore (http://store2.asco.org/), to name just a few.

For clinicianas who prefer to learn on the go, on their smartphones and/or tablets, ASCO University has mobile learning options available as well (http://university.asco.org/mobile-learning-0), including self-assessment questions that are sent to the subscriber’s email account after signing up for one of the *Daily Medical Education* courses featured on the Mobile Learning web page.

Some ASCO University content is also available on iTunes U. Links to available content are found on the Mobile Learning web page or one may go directly to iTunes U and search for content using "ASCO University" as the search term.

## Conclusion

Given the broad scope of educational programs and content available on the ASCO University website, it is worthwhile for clinicians to take some time to browse through the website and see all that it has to offer. A robust search engine is part of each page of the ASCO University website and can direct clinicians to desired content. Much of the content on this website can be used by advanced practice providers in oncology to meet their ongoing educational needs; for courses that are offered for a fee, discounts may be available to ASCO members. Information on becoming a member of ASCO can be found at http://benefits.asco.org/.

